# Acute Onset Polymyositis after Prolactinoma Extirpation

**DOI:** 10.1155/2014/329059

**Published:** 2014-11-06

**Authors:** Juan Jakez-Ocampo, Yemil Atisha-Fregoso, Luis Llorente

**Affiliations:** ^1^Department of Immunology and Rheumatology, Instituto Nacional de Ciencias Médicas y Nutrición Salvador Zubirán, Vasco de Quiroga 15, Tlalpan, 14000 Mexico City, Mexico; ^2^Medicine Direction, Instituto Nacional de Ciencias Médicas y Nutrición Salvador Zubirán, Vasco de Quiroga 15, Tlalpan, 14000 Mexico City, Mexico

## Abstract

Hyperprolactinemia has been related to autoimmune diseases. Herein, we describe a case of a female with a prolactin producer pituitary macroadenoma who developed severe polymyositis one month after its removal. The patient had very high levels of CPK and muscle biopsy showed remarkable inflammatory infiltration. Steroid therapy was followed with total recovery. To the best of our knowledge, this is the first case reported of acute polymyositis after pituitary macroadenoma exeresis.

## 1. Introduction 

Polymyositis is a chronic autoimmune disease that causes proximal muscle weakness as well as remarkable functional limitation. Its aetiology is unknown; it has been suggested, however, that prolactin could play a role in its development. This is based on the fact that high levels of this hormone have been found in up to 25% of patients with polymyositis [1]; it has also been shown that patients with hyperprolactinemia have one or more autoantibodies in their serum [2]. In this report we describe the case of a patient with a prolactin-producing pituitary macroadenoma who, one month after its extirpation, presented with polymyositis.

## 2. Case Report

A 39-year-old woman without any relevant background started having an intermittent headache in 2008, which improved with common analgesics. Six months later, she noticed amenorrhea, with repeated negative pregnancy tests. Her gynaecologist ordered both blood tests and head magnetic resonance imaging (MRI) that showed hyperprolactinemia and a pituitary microadenoma. He prescribed cabergoline 1.5 mg twice a week with a good response for two years, when the patient noticed visual deficit and increased headache intensity. On admission to our hospital, the only alteration found on physical examination was bitemporal hemianopsia. Blood tests reported haemoglobin of 14.8 g/dL (13–15.7), WBC of 6.7 K/*μ*L (4–12), platelets of 287 K/*μ*L (150–450), ASAT of 37 U/mL (13–39), ALAT of 35 U/ML (7–52), glucose of 98 mg/dL (65–100), creatinine of 0.7 mg/dL (0.6–1.2), and prolactin of 75 ng/mL (2–29). Head MRI now showed a pituitary macroadenoma which was removed by endoscopic transnasal exeresis. After this procedure, prolactin levels returned to normal values.

Just one month after surgery, the patient started with progressive muscle weakness that became worse in the following two weeks until it left her prostrate in bed. A heart rate of 120 beats/min, a respiratory rate of 20/min, blood pressure of 90/50 mm Hg, and temperature of 37°C were found upon arrival to the emergency room. She was extremely weak, to the extent that she was able to neither keep her head up nor swallow. Blood tests now showed haemoglobin of 9.7 g/dL (13.0–15.7), WBC of 9.8 K/*μ*L (4–12), and platelets of 287 K/*μ*L (150–450). Antinuclear antibodies were positive with speckled pattern (1 : 320) and anti-Jo1 antibodies were negative at 2.1 U/mL (<6.5). No other antibodies were tested. CPK was 7377 U/mL (37–177), ASAT was 891 U/mL (13–39), and ALAT was 609 U/mL (7–52). Respiratory acidosis was also detected with pCO2 of 46 mm Hg in arterial blood which necessitated the patient to be put under noninvasive positive pressure ventilation. Muscle MRI showed remarkable inflammatory alteration with diffuse bilateral myofibrillar intensity of all muscle groups evaluated, from the pelvis to the distal quadriceps. Muscle biopsy of vastus lateralis demonstrated notable inflammatory infiltration with atrophy, degenerative changes, and myocitolysis.

Taking the above data into account, treatment was started with 8 mg of IV dexamethasone every 24 hrs, which resulted in a progressive decrease of serum's CPK levels until their normalisation; the patient, however, had to be kept on totally enteric nutrition through a gastrostomy for one month, after which she recovered her deglutition ability. At the time of writing, the patient is completely asymptomatic and is under treatment with 100 mg azathioprine.

## 3. Discussion

This case shows a clear temporal relationship between the extraction of the prolactinoma and the onset of disease. Is it possible, however, that, in addition to a simple association, there could be a causal relationship between these phenomena?

There is a vast amount of information regarding the presence of hyperprolactinemia in rheumatic autoimmune diseases [3]. This is particularly so in systemic lupus erythematosus (SLE), where it is thought that prolactin may even play a role in its pathogenesis [2]. A likely mechanism involves the adaptive immune response, mainly by alterations in B cell tolerance in three different ways: by the impairing of B cell receptor mediated clonal deletion; by the deregulation receptor editing; and by decreasing the threshold for activation of anergic B cells [4]. At the molecular level, prolactin upregulates expression of costimulatory molecules such as CD40, CD80, and CD86 [5] and also upregulates Bcl-2 in B cells. In fact, it has been put forward that the latter effect could favor the survival of autoreactive cells, rescuing them from negative selection [6]. Besides these effects, prolactin requires a genetic susceptibility background to produce autoimmunity [7].

Interestingly, prolactin concentrations at slightly higher levels than the physiological range but not those vastly superior induce an increased production of IgG by mononuclear cells from SLE patients [8], thus creating a prolactin threshold for its contribution to autoimmunity.

Thus, even though it is possible that the chronic exposure to slightly higher levels of prolactin in a genetically susceptible individual could have contributed to the onset of polymyositis, the actual manifestation of the disease did not take place until the removal of the tumor, which begs an obvious question: why was the disease dormant until precisely that moment? In this regard, dopamine agonists have shown a therapeutic effect in SLE and, less consistently, in RA and psoriasis [9, 10]. It is also important to point out that, in one study with SLE patients, a relapse into the disease was suffered by all patients after the withdrawal of bromocriptine [11].

Hence, the possibility exists that, in this case, the presence of cabergoline could have acted as therapy for an incipient autoimmune disease. Even though dopamine-agonist could have helped to maintain a quiescent state of the disease, both tumor removal and treatment withdrawal were probably the trigger for the full-blown manifestation of the disease. Certainly, this speculation, although undoubtedly interesting, is based on the role of prolactin in other autoimmune diseases, whilst not in polymyositis.

Be that as it may, this case is interesting inasmuch as it represents the first description of acute onset polymyositis after removal of a pituitary macroadenoma in the English literature.
